# Unveiling the environmental significance of acetylperoxyl radical: Reactivity quantification and kinetic modeling

**DOI:** 10.1093/pnasnexus/pgae330

**Published:** 2024-08-07

**Authors:** Junyue Wang, Thomas Schaefer, Aliaksandra Lisouskaya, Daniele S Firak, Xiaoyue Xin, Lingjun Meng, Hartmut Herrmann, Virender K Sharma, Ching-Hua Huang

**Affiliations:** School of Civil and Environmental Engineering, Georgia Institute of Technology, 200 Bobby Dodd Way NW, Atlanta, GA 30332, USA; Atmospheric Chemistry Department (ACD), Leibniz Institute for Tropospheric Research (TROPOS), Permoserstraße 15, 04318 Leipzig, Germany; Radiation Laboratory, University of Notre Dame, 102 Radiaiton Research Building, Notre Dame, IN 46556, USA; Atmospheric Chemistry Department (ACD), Leibniz Institute for Tropospheric Research (TROPOS), Permoserstraße 15, 04318 Leipzig, Germany; School of Civil and Environmental Engineering, Georgia Institute of Technology, 200 Bobby Dodd Way NW, Atlanta, GA 30332, USA; School of Civil and Environmental Engineering, Georgia Institute of Technology, 200 Bobby Dodd Way NW, Atlanta, GA 30332, USA; Atmospheric Chemistry Department (ACD), Leibniz Institute for Tropospheric Research (TROPOS), Permoserstraße 15, 04318 Leipzig, Germany; Department of Environment and Occupational Health, School of Public Health, Texas A&M University, 212 Adriance Lab Road, College Station, TX 77843, USA; School of Civil and Environmental Engineering, Georgia Institute of Technology, 200 Bobby Dodd Way NW, Atlanta, GA 30332, USA

**Keywords:** radical chemistry, organic peroxyl radical, laser flash photolysis, pulse radiolysis, advanced oxidation process

## Abstract

Acetylperoxyl radical (CH_3_C(O)OO^•^) is among highly reactive organic radicals which are known to play crucial roles in atmospheric chemistry, aqueous chemistry and, most recently, peracetic acid (PAA)-based advanced oxidation processes. However, fundamental knowledge for its reactivity is scarce and severely hampers the understanding of relevant environmental processes. Herein, three independent experimental approaches were exploited for revelation and quantification of the reaction rates of acetylperoxyl radical. First, we developed and verified laser flash photolysis of biacetyl, ultraviolet (UV) photolysis of biacetyl, and pulse radiolysis of acetaldehyde, each as a clean source of CH_3_C(O)OO^•^. Then, using competition kinetics and selection of suitable probe and competitor compounds, the rate constants between CH_3_C(O)OO^•^ and compounds of diverse structures were determined. The three experimental approaches complemented in reaction time scale and ease of operation, and provided cross-validation of the rate constants. Moreover, the formation of CH_3_C(O)OO^•^ was verified by spin-trapped electron paramagnetic resonance, and potential influence of other reactive species in the systems was assessed. Overall, CH_3_C(O)OO^•^ displays distinctively high reactivity and selectivity, reacting especially favorably with naphthyl and diene compounds (*k* ∼ 10^7^–10^8^ M^−1^ s^−1^) but sluggishly with N- and S-containing groups. Significantly, we demonstrated that incorporating acetylperoxyl radical-oxidation reactions significantly improved the accuracy in modeling the degradation of environmental micropollutants by UV/PAA treatment. This study is among the most comprehensive investigation for peroxyl radical reactivity to date, and establishes a robust methodology for investigating organic radical chemistry. The determined rate constants strengthen kinetic databases and improve modeling accuracy for natural and engineered systems.

**Significance Statement**

Organic radicals are ubiquitous in the atmosphere, aqueous environments, and living cells, yet their reactivity has been scarcely studied. Herein, we comprehensively investigated the reactivity of acetylperoxyl radical (CH_3_C(O)OO^•^, which has been suggested among the most oxidative organic radicals), with structurally diverse organic compounds using laser flash photolysis, pulse radiolysis and ultraviolet photoreactor approaches. This study not only unveils the high reactivity and selectivity of acetylperoxyl radical, facilitating more accurate modeling and elucidating their importance in environmental and catalytic oxidation processes, but also establishes a robust strategy for future research on organic radicals.

## Introduction

Organic peroxyl radicals, commonly produced by oxygen (O_2_) addition onto carbon-centered radicals, are ubiquitous in natural environments and living cells (1). Although most peroxyl radicals lack reactivity and undergo fast self-decay, acyl peroxyl radicals (R-C(O)OO^•^) are among the most reactive and stable organic radicals (2–4). For instance, acetylperoxyl radical (CH_3_C(O)OO^•^) is among the most long-lived (i.e. a first-order self-decay rate at 1.82 s^−1^) and reactive organic radicals with a standard redox potential at ∼1.6 V (vs normal hydrogen electrode) (5, 6) Nonetheless, previous studies have mainly focused on inorganic radicals (e.g. atomic O, ^•^OH, and ^•^Cl), while the kinetic information of those reactive organic radicals has received relatively limited exploration.

**Fig. 1.**
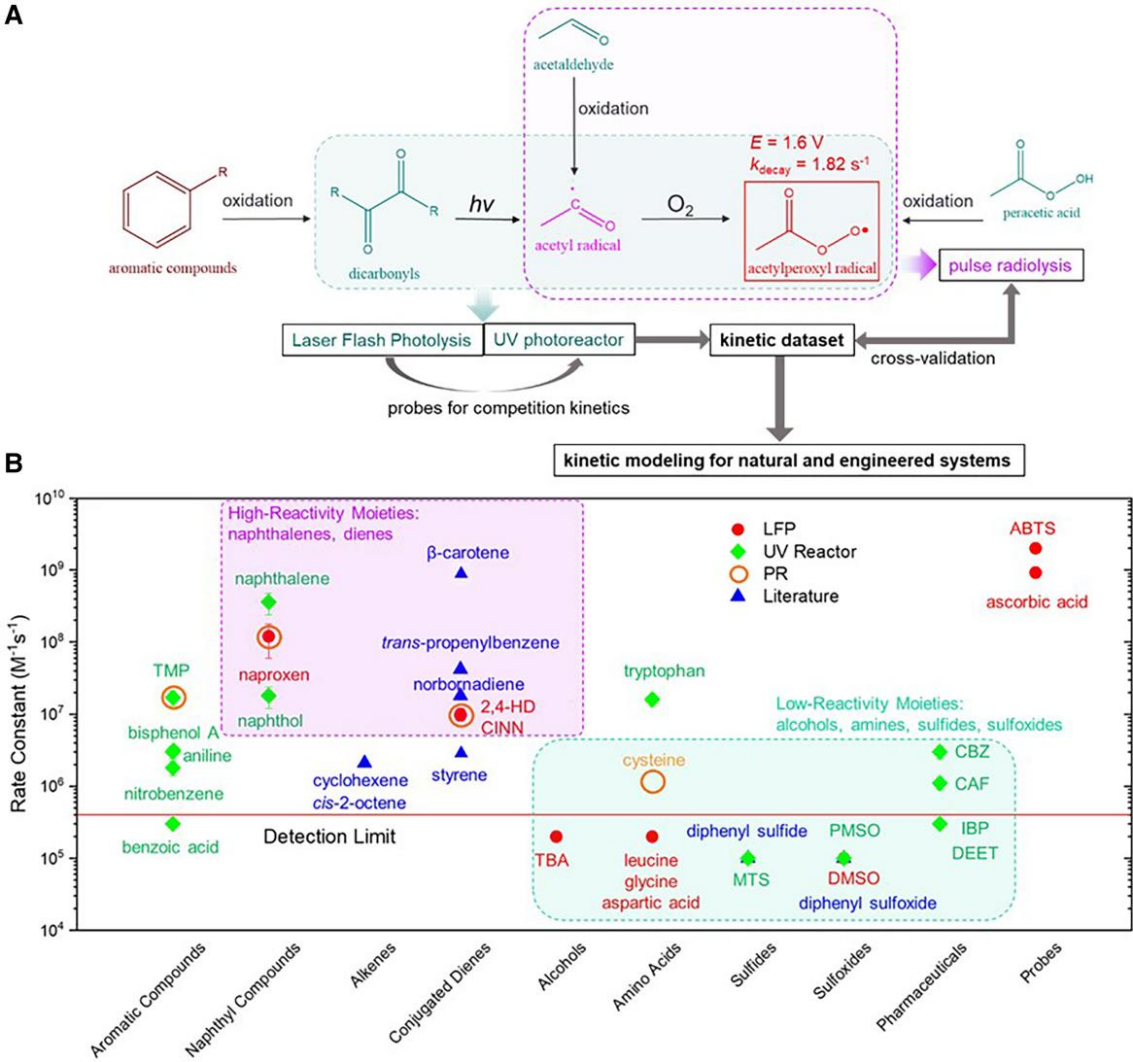
Generation pathways of acetylperoxyl radical and the methodology summary for quantifying the reactivity of acetylperoxyl radical (A), second-order rate constants between acetylperoxyl radical and organic compounds (B).

Under aerobic conditions, CH_3_C(O)OO^•^ could be generated from (i) photolysis of dicarbonyl compounds (7–9), (ii) oxidation of aldehydes (10, 11), and (iii) oxidation of peracetic acid (PAA, CH_3_C(O)OOH) (Fig. 1A) (12–15). Owing to its high redox potential and relatively higher steady-state concentration, CH_3_C(O)OO^•^ has essential roles in environmental and biochemical processes. For example, its reactions with nitrogen oxides (NO_x_), dimethyl sulfide, and sulfur dioxide (SO_2_) could significantly affect the N and S cycles in the atmosphere (1, 16). The reactions with biomolecules may lead to cellular damage or loss of antioxidant capacity (17, 18). Moreover, CH_3_C(O)OO^•^ has been recently extensively postulated in PAA-based catalytic oxidation and advanced oxidation processes (AOPs), where PAA is activated by external energy, trace metals, or heterogeneous catalysts for (waste)water decontamination and purification. CH_3_C(O)OO^•^, generated by single-electron transfer oxidation of PAA (Eqs. 1 and 2), is an important candidate for the degradation of organic contaminants during PAA activation by ultraviolet (UV) (12, 19, 20), Fe species (14, 15, 21), Co species (13, 22–24), and other metal species [e.g. Mn(II) (25), Ru(III) (26), and Cr(III) (27)]. Additionally, CH_3_C(O)OO^•^ is also a candidate reactive species during water decontamination by UV/diketone AOPs (28).


(1) $$M^{(n\;+\;1)+\;}+CH_{3}C(O)\mathrm{OOH}\to M^{n\;+\;}+CH_{3}C(O)OO^{∙}+H^{+}\;$$

(2) OH∙+CH3C(O)OOH→OH−+CH3C(O)OO∙

To date, however, there is a dearth of information regarding the reactivity of CH_3_C(O)OO^•^, which hinders the delineation of its contribution in natural geochemical and biochemical cycles, as well as engineered AOP systems. For instance, the contribution of CH_3_C(O)OO^•^ in the PAA- or diketone-based water decontamination processes has only been indirectly inferred or roughly estimated, rather than quantitatively examined due to the inability to distinguish CH_3_C(O)OO^•^ from other radicals and high-valent metal species co-generated in the AOP systems (12–15, 26, 29). Therefore, an unambiguous, comprehensive investigation on the reactivity of CH_3_C(O)OO^•^ is urgently needed.

Herein, we developed a laser flash photolysis (LFP) method using 2,3-butanedione (hereinafter referred to as biacetyl) in aerobic solutions as a clean source to predominantly generate CH_3_C(O)OO^•^, and 2,2′-azino-bis(3-ethylbenzothiazoline-6-sulfonic acid) (ABTS) as the colorimetric probe, to determine the rate constants between CH_3_C(O)OO^•^ and compounds such as naproxen (NPX), *trans*-cinnamic acid (CINN), and others. Then, NPX and CINN were employed as probes for competition kinetics in a UV/biacetyl reactor, where the rate constants of CH_3_C(O)OO^•^ with various structurally diverse organic compounds were comprehensively investigated by tracking their degradation during the UV/biacetyl AOP (SI Appendix, Table S1). Furthermore, the key rate constants were cross-validated by pulse radiolysis (PR) of acetaldehyde, another clean source of CH_3_C(O)OO^•^ (Fig. 1A). Overall, this study not only built a kinetic dataset (Fig. 1B) of CH_3_C(O)OO^•^, but also established a systematic approach for investigating CH_3_C(O)OO^•^ that could be further utilized for studying other organic radicals.

## Results and discussions

### Reactive species in LFP of biacetyl

**Fig. 2.**
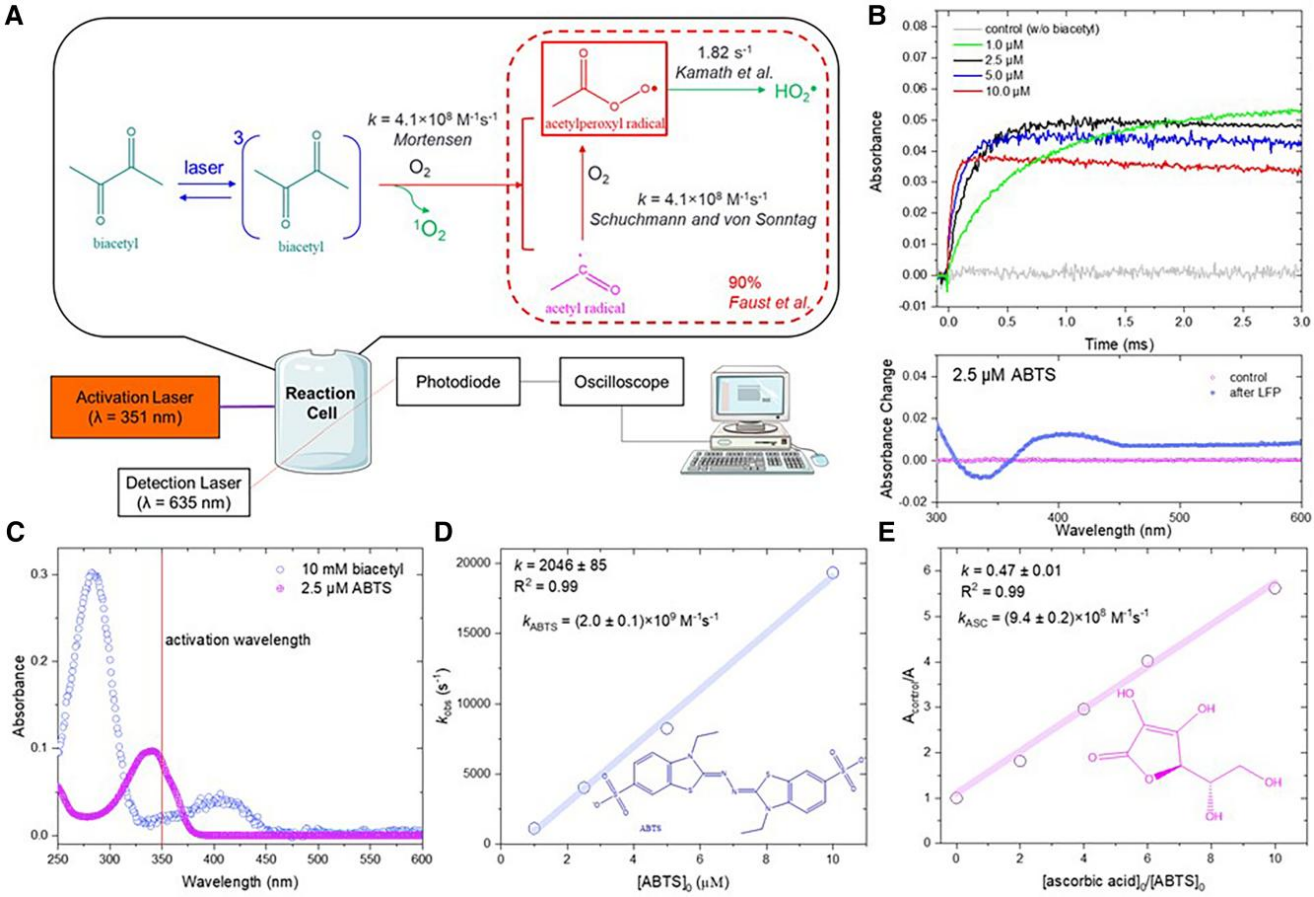
The experimental setup and the mechanism for acetylperoxyl radical generation by LFP of biacetyl (A), effect of initial ABTS concentration on ABTS oxidation during LFP of biacetyl (represented by ABTS^•+^ formation recorded at 635 nm) (B), absorbance spectra of biacetyl and ABTS (C), linear relationship between initial ABTS concentration and ABTS^•+^ formation rate (D), linear relationship between ascorbic acid/ABTS molar ratio and relative ABTS^•+^ formation (E). Experimental conditions: [biacetyl]_0_ = 10 mM, pH_0_ = 5.7 (not buffered); for (D), [ABTS]_0_ = 2.5 µM.

The setup of the LFP system is illustrated in Fig. 2A (30). The radical precursor, biacetyl (10 mM), was photolyzed with an excimer laser at λ = 351 nm to generate CH_3_C(O)OO^•^ in the presence of dissolved oxygen (∼ 2.6 × 10^−4^ M, measured by a DO meter) (Fig. 2A, see later discussion). ABTS, whose reaction with CH_3_C(O)OO^•^ has been studied previously [Eq. 3; (31, 32)], was used as the probe for competition kinetics.


(3) $$\begin{array}{rl} \mathrm{ABTS}+ & CH_{3}C(O)OO^{∙}\to CH_{3}C(O)OO^{-}\\ & +\mathrm{ABT}S^{∙+}\;k=(1.8-2.0)\times 10^{9}\;M^{-1}s^{-1} \end{array}$$

The absorbance spectra of reaction solutions before and after LFP experiments were measured by a UV–vis spectrophotometer. Obvious absorbance change was observed after LFP (Fig. 2B), with the maximum decrease at ∼ 340 nm and maximum increase at ∼ 415 nm, clearly indicating a conversion from ABTS (peak absorbance: ε_340_ _nm_ = 3.66 × 10^4^ M^−1^ cm^−1^; Fig. 2C) to ABTS^•+^ radical (peak absorbance: ε_415_ _nm_ = 3.4 × 10^4^ M^−1^ cm^−1^; SI Appendix, Figure S2). The ABTS^•+^ once produced was stable in the next few millisecond and was measured at 635 nm versus time (Fig. 2B, SI Appendix). Notably, the laser light at 351 nm did not lead to ABTS photolysis or ABTS^•+^ generation (control in Fig. 2B), suggesting that ABTS was oxidized by reactive species generated by laser-activated biacetyl. Preliminary experiments showed 248 nm laser not suitable due to photodegradation of ABTS.

The photolysis of biacetyl has been extensively studied and the pathway is depicted as Fig. 2A (7, 29, 33). The triplet biacetyl undergoes C–C bond cleavage between two carbonyl groups to generate two CH_3_C(O)^•^ with a ∼ 90% yield (reported at 313 nm) (7), which reacts with O_2_ at 4.1 × 10^8^ M^−1^ s^−1^ to generate CH_3_C(O)OO^•^ (32). In addition, dissolved O_2_ scavenges triplet biacetyl at 4.1 × 10^8^ M^−1^ s^−1^, producing either singlet oxygen (^1^O_2_) or CH_3_C(O)^•^ + CH_3_C(O)OO^•^ (14, 34). Due to the low reactivity of ^1^O_2_ and CH_3_C(O)^•^, the previous studies attributed all ABTS oxidation to CH_3_C(O)OO^•^ (31, 33). Meanwhile, CH_3_C(O)OO^•^ may undergo a slow self-decomposition (1.82 s^−1^) to generate superoxide radicals (HO_2_^•^/O_2_^•−^) (Fig. 2A). Notably, 40% of the CH_3_C(O)^•^ may be hydrated to produce CH_3_C(OH)_2_^•^, and subsequently CH_3_C(OH)_2_OO^•^, which is easily decomposed to give O_2_^•−^ (32). The hydration of CH_3_C(O)OO^•^ was not expected (32). Hence, the candidate reactive species for ABTS oxidation during LFP of biacetyl include triplet biacetyl, ^1^O_2_, CH_3_C(O)^•^, CH_3_C(O)OO^•^, and HO_2_^•^/O_2_^•−^. Their potential roles are addressed one by one as below.

^1^O_2_ is a weak oxidant that could not oxidize various susceptible organic compounds [e.g. tetramethyl-*p*-phenylenediamine (TMPD) (8)], hence its role in oxidizing the recalcitrant contaminants is minor (35, 36). Furthermore, the lifetime of ^1^O_2_ is reported to be ∼ 3.8 µs in H_2_O (37, 38), which is definitely too short to be responsible for the much longer term ABTS oxidation that lasted for milliseconds (Fig. 2B).

Recent literature showed the triplet biacetyl also exhibited oxidation capacity for some organic compounds including carbamazepine (CBZ) (29) and β-carotene (33), evidenced by their nonnegligible removal in the absence of oxygen (O_2_), which is critical for the formation of CH_3_C(O)OO^•^. However, triplet organic matter is always rapidly quenched by the surrounding solvent and/or O_2_, hence their lifetime and oxidation kinetics typically last for only microseconds in the presence of O_2_ (39, 40). Previous literature has confirmed that, despite its oxidation contribution in anoxic conditions, triplet biacetyl could not contribute to oxidation in the presence of O_2_ due to the fast quenching reaction that leads to the formation of either ^1^O_2_ or CH_3_C(O)^•^ + CH_3_C(O)OO^•^. For example, Mortensen *et al.* (33) distinguished triplet biacetyl and CH_3_C(O)OO^•^ by their different oxidation products of β-carotene and confirmed the negligible role of triplet biacetyl in aerated solution. Darmanyan *et al.* (34) observed the rapid quenching of triplet biacetyl by O_2_ directly by the transient absorption spectra changes in the solution in equilibrium with air. Jin *et al.* (41) also found the triplet state acetylacetone (another simple diketone) had a lifetime and oxidation duration at microsecond level. Furthermore, we estimated the lifetime of triplet biacetyl to be only 6.1 µs in aerobic solution due to the quenching by O_2_ ([O_2_] = ∼2.6 × 10^−4^ M, *k*_O2_ = 4.1 × 10^8^ M^−1^ s^−1^) (14, 34), hence triplet biacetyl is not responsible for the millisecond-long oxidation of ABTS.

**Fig. 3.**
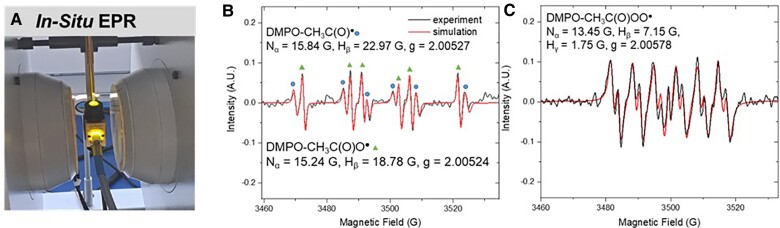
The setup of in situ spin-trapped EPR (A), EPR spectra of UV/biacetyl in water (B) or in TBA solvent (C). Conditions: [biacetyl] = 1 mM, [DMPO] = 2 mM, irradiation time = 734 s.

HO_2_^•^/O_2_^•−^, produced from self-decomposition of CH_3_C(O)OO^•^ and its hydrated form (CH_3_CH(OH)_2_OO^•^) is another potential oxidant in our system, with a lifetime at microsecond level (32). If HO_2_^•^/O_2_^•−^ is responsible for ABTS oxidation, a pH decrease should lead to protonation of O_2_^•−^ [p*K*_a_ = 4.8 (42)] and accelerate ABTS^•+^ formation. However, we decreased the initial pH from 5.7 to 3.2 and found a negligible change in ABTS^•+^ formation kinetics (SI Appendix, Figure S3). Additionally, neither the signal for HO_2_^•^/O_2_^•−^ or HO^•^ adduct with 5,5-dimethyl-1-pyrroline N-oxide (DMPO) was observed in spin-trapped electron paramagnetic resonance (EPR) (Fig. 3), hence we expect a negligible contribution of HO_2_^•^/O_2_^•−^ in the system. More importantly, we used PR of acetaldehyde to confirm that HO_2_^•^/O_2_^•−^, as well as CH_3_CO^•^, could hardly react with ABTS (see later Discussion). Therefore, the only candidate for ABTS oxidation in the LFP system was CH_3_C(O)OO^•^.

### Observation of acetylperoxyl radicals by spin-trapped EPR

In situ EPR was harnessed for direct identification of CH_3_C(O)OO^•^ (Fig. 3A, SI Appendix). We could not detect CH_3_C(O)OO^•^ in water samples due to instability of the adduct of CH_3_C(O)OO^•^ with DMPO (Fig. 3B), similar to previous research (43). Therefore, we employed *tert*-butyl alcohol (TBA), which has negligible reactivity toward CH_3_C(O)OO^•^, as the medium to stabilize the DMPO-peroxyl adduct without quenching the radical (43). During the in situ photolysis of biacetyl, DMPO-CH_3_C(O)OO^•^ was accumulated, successfully identified, and simulated with the conditions marked in Fig. 3C, further confirming its critical role in the UV/biacetyl system. Notably, our EPR pattern was consistent with Hoshino *et al.*'s (8) result for aerobic biacetyl photolysis in benzene solution. Noteworthy, DMPO's adducts with ^•^OH or HO_2_^•^/O_2_^•−^, and its oxidation product by ^1^O_2_ (5,5-dimethylpyrrolidone-2(2)-oxyl-(1) (DMPOX) and DMPO-^•^OH) (44) were not observed in either deionized (DI) water or TBA solutions, indicating the minor roles of these reactive species during continuous irradiation of biacetyl.

### The rate constant between ABTS and acetylperoxyl radical

As ABTS oxidation could be primarily attributed to CH_3_C(O)OO^•^, the rate constant between ABTS and CH_3_C(O)OO^•^ could be first determined by modeling the pseudo-first-order ABTS^•+^ buildup rate at different initial ABTS concentrations (Eqs. 4 and 5).


(4) $$\begin{array}{rl} \frac{d{\left[\mathrm{ABT}S^{∙+}\right]}_{t}}{\mathrm{dt}} & =-\frac{d{\left[{\mathrm{CH}}_{3}C(O)OO^{∙}\right]}_{t}}{\mathrm{dt}}\\ & =k_{\mathrm{ABTS}}[CH_{3}C(O)OO^{∙}{]}_{t}[\mathrm{ABTS}{]}_{t} \end{array}$$

(5) $$\;\int \frac{d{\left[{\mathrm{CH}}_{3}C(O)OO^{∙}\right]}_{t}}{{\left[{\mathrm{CH}}_{3}C(O)OO^{∙}\right]}_{t}}=-\int k_{\mathrm{ABTS}}[\mathrm{ABTS}{]}_{t}\mathrm{dt}=\ln\frac{{\left[{\mathrm{CH}}_{3}C(O)OO^{∙}\right]}_{t}}{{\left[{\mathrm{CH}}_{3}C(O)OO^{∙}\right]}_{0}}$$

$k_{\mathrm{ABTS}}$ is the second-order rate constant between ABTS and CH_3_C(O)OO^•^ (in M^−1^ s^−1^); *t* is reaction time after activation by the laser pulse (in s); [ABTS]*_t_* and [CH_3_C(O)OO^•^]*_t_* are the concentrations of ABTS and CH_3_C(O)OO^•^ at the particular reaction time *t* (in M), respectively; [CH_3_C(O)OO^•^]_0_ is initial concentration of CH_3_C(O)OO^•^ generated by the pulse laser (in M). As ABTS was in excess of the produced CH_3_C(O)OO^•^, we could assume the ABTS concentration remained close to the initial concentration. Considering the 1:1 stoichiometry between CH_3_C(O)OO^•^ and ABTS (Eq. 3), $k_{\mathrm{ABTS}}$ can be calculated by Eq. 6.


(6) $$\begin{array}{rl} \ln\frac{{\left[{\mathrm{CH}}_{3}C(O)OO^{∙}\right]}_{t}}{{\left[{\mathrm{CH}}_{3}C(O)OO^{∙}\right]}_{0}} & =\ln\frac{{\left[\mathrm{ABT}S^{∙+}\right]}_{\mathrm{final}}-{\left[\mathrm{ABT}S^{∙+}\right]}_{t}}{{\left[\mathrm{ABT}S^{∙+}\right]}_{\mathrm{final}}}\\ & =-k_{\mathrm{ABTS}}[\mathrm{ABTS}{]}_{0}t=\ln\frac{A_{\mathrm{final}}-A_{t}}{A_{\mathrm{final}}} \end{array}$$

[ABTS^•+^]_final_ is the final ABTS^•+^ concentration after its formation reaches the plateau, which equals to [CH_3_C(O)OO^•^]_0_; *A*_final_ is the final absorbance at 635 nm (i.e. when ABTS^•+^ formation reaches the plateau), and *A_t_* is the absorbance at time *t*. Preliminary tests have shown that the photolysis of biacetyl and the selected compounds could not produce signal at 635 nm, hence the *A_t_* and *A*_final_ should be linearly proportional to the corresponding ABTS^•+^ concentration.

As shown in Fig. 2B and D, the buildup of 635 nm signal (indicating ABTS^•+^ formation) was faster with increasing ABTS concentration, consistent with the kinetic calculation. On the other hand, *A*_final_, indicating [ABTS^•+^] at the plateau and the total amount of photo-generated CH_3_C(O)OO^•^, decreased with increasing ABTS concentration. This is due to the competitive light absorbance of ABTS with biacetyl, where ABTS dominated the overall light screening at 351 nm despite its much lower concentration (Fig. 2C). According to Eq. 6, *k*_ABTS_ was determined to be (2.0 ± 0.1) × 10^9^ M^−1^ s^−1^, by linear regression of the pseudo-first-order rate constants for signal buildup against initial ABTS concentrations (Figs. 2D and 1B, SI Appendix, Table S1). This rate constant is in excellent agreement with the reported values, confirming the accuracy of our analytical methods (31, 32). Notably, the oxidation of ABTS lasted for several milliseconds, indicating a relatively long lifetime of CH_3_C(O)OO^•^ and ruling out the contribution of other transient intermediates (e.g. triplet biacetyl, ^1^O_2_) with microsecond-lifetime.

### Competition kinetics study in LFP of biacetyl

In the competition kinetics experiments, biacetyl, ABTS, and compound X (at different concentrations) were mixed together and underwent pulse laser activation, where the photo-generated CH_3_C(O)OO^•^ was consumed by either compound X or ABTS (Eqs. 7 and 8).


(7) $$\begin{array}{rl} \int d{\left[\mathrm{ABT}S^{∙+}\right]}_{t}=\Delta [\mathrm{ABT}S^{∙+}]= & \;-\Delta [\mathrm{ABTS}]=k_{\mathrm{ABTS}}[\mathrm{ABTS}{]}_{0}\\ & \int {\left[{\mathrm{CH}}_{3}C(O)OO^{∙}\right]}_{t}\mathrm{dt} \end{array}$$

(8) $$\int d[X{]}_{t}=\Delta [X]=-k_{X}[X{]}_{0}\int [{\mathrm{CH}}_{3}C(O)OO^{∙}{]}_{t}\mathrm{dt}$$

$k_{X}$ is the second-order rate constant between selected compound X and CH_3_C(O)OO^•^ (in M^−1^ s^−1^); Δ[X], Δ[ABTS], Δ[ABTS^•+^] are the concentration changes of X, ABTS, and ABTS^•+^, respectively, after the ABTS oxidation reaches the plateau (in M). Note that the change in overall absorbance at 351 nm was negligibly affected by selected compounds, hence the addition of compound X could not affect CH_3_C(O)OO^•^ production by light-shielding or producing additional reactive species. In other words, [CH_3_C(O)OO^•^]_0_ remained at the same value regardless of the addition of compound X, and the difference between [CH_3_C(O)OO^•^]_0_ and [ABTS^•+^]_final_ should be totally attributed to the consumption by compound X (i.e. Δ[X]) (Eq. 9).


(9) $$\begin{array}{rl} \frac{\Delta [X]}{\Delta [\mathrm{ABTS}]} & =\frac{k_{X}{\left[X\right]}_{0}}{k_{\mathrm{ABTS}}{\left[\mathrm{ABTS}\right]}_{0}}=\;\frac{{\left[{\mathrm{CH}}_{3}C(O)OO^{∙}\right]}_{0}-{\left[\mathrm{ABT}S^{∙+}\right]}_{\mathrm{final}}}{{\left[\mathrm{ABT}S^{∙+}\right]}_{\mathrm{final}}}\\ & =\frac{A_{\mathrm{control}}}{A_{X}}-1 \end{array}$$

*A*_control_ is the final absorbance (indicating ABTS^•+^ formation) without compound X; and *A*_X_ is the final absorbance with compound X.

The reactivity of CH_3_C(O)OO^•^ toward ascorbic acid has been investigated in previous studies (31, 32). Hence, we tested the reactivity of ascorbic acid by competition kinetics with ABTS in the same LFP setup (Eqs. 8 and 9) to further validate our approach. *A*_final_, indicating ABTS^•+^ formation, decreased remarkably with increasing ascorbic acid concentration, suggesting that the ABTS oxidation was inhibited due to the coexistent ascorbic acid competing for CH_3_C(O)OO^•^ (Fig. 2E). Through a linear regression according to Eq. 9, the rate constant between ascorbic acid and CH_3_C(O)OO^•^ was determined to be (9.4 ± 0.2) × 10^8^ M^−1^ s^−1^ (Fig. 2E, SI Appendix, Table S1), concurring with previous studies and confirming the reliability of our method (32).

Subsequently, the reactivity of CH_3_C(O)OO^•^ toward NPX, CINN, and 2,4-hexadiene (2,4-HD) was studied in the LFP system by competition kinetics with ABTS. Compared to ABTS, these compounds have negligible competitive light absorption with biacetyl at 351 nm, and their reactivity to CH_3_C(O)OO^•^ is lower than that of ABTS but spans a measurable range for the competition kinetics method (Eq. 9). CH_3_C(O)OO^•^ exhibited the highest reactivity with NPX at (1.2 ± 0.6) × 10^8^ M^−1^ s^−1^, followed by CINN and 2,4-HD at ∼10^7^ M^−1^ s^−1^ (Fig. 1B, SI Appendix, Table S1, Figure S4) (8, 45). On the contrary, the addition of TBA, dimethyl sulfoxide (DMSO), leucine, glycine, and aspartic acid had a negligible impact on ABTS oxidation, hence their rate constants with CH_3_C(O)OO^•^ were too low to determine (Fig. 1B, SI Appendix, Table S1, Figure S5), indicating the low reactivity of CH_3_C(O)OO^•^ toward aliphatic alcohols, amino acids, and sulfoxides [reported (45)].

### Competition kinetics study in a UV reactor with biacetyl

To investigate the rate constants between less reactive compounds with CH_3_C(O)OO^•^, we compared the degradation of selected organic compounds versus suitable probes during UV/biacetyl AOP in a bench-scale UV (254 nm) reactor, which enables continuous CH_3_C(O)OO^•^ generation and long-term monitoring of compound degradation. Biacetyl was continuously activated by UV_254_ irradiation following the photochemical pathways in Fig. 2A, generating CH_3_C(O)OO^•^ as the sole reactive species for degradation of selected organic compounds (in addition to direct photolysis degradation). Therefore, the difference between degradation rates during direct photolysis (without biacetyl addition) and UV/biacetyl could be attributed to the oxidation by CH_3_C(O)OO^•^, and the oxidation rate should be linearly proportional to their second-order rate constants with CH_3_C(O)OO^•^ (Eq. 10).


(10) $$\frac{k_{\mathrm{obs},\mathrm{probe}}-k_{\mathrm{UV},\mathrm{probe}}}{k_{\mathrm{obs},X}-k_{\mathrm{UV},X}}=\frac{k_{\mathrm{probe}}}{k_{X}}\;$$

$k_{\mathrm{obs},\mathrm{probe}}$ and $k_{\mathrm{obs},X}\;$ are the observed pseudo-first-order rate constants for the degradation of the probe compound (whose reactivity has been quantified) and compound X, respectively (in min^−1^); $\;k_{\mathrm{UV},\mathrm{probe}}$ and $k_{\mathrm{UV},X}\;$ are their direct photolysis rate constants (in min^−1^); $k_{\mathrm{probe}}$ and $k_{X}\;$ are the second-order rate constants between CH_3_C(O)OO^•^ and the probe and compound X, respectively (in M^−1^ s^−1^). NPX and CINN, which can be easily measured by high-performance liquid chromatography and have been investigated by LFP, were selected as probes for the competition kinetics study in the UV reactor.

Firstly, the method was validated by comparing the degradation of NPX and CINN in the UV reactor (SI Appendix, Figure S7). As calculated by Eq. 10, the observed pseudo-first-order oxidation rate constant $\left(k_{\mathrm{obs}-}k_{\mathrm{UV}}\right)$ of NPX was about 12 times higher than that of CINN, very close to the ratio of their second-order rate constants with CH_3_C(O)OO^•^ obtained from the LFP system (SI Appendix, Table S1). Subsequently, the rate constants of CH_3_C(O)OO^•^ with more organic compounds were determined by competition kinetics with either NPX or CINN in the UV reactor (SI Appendix, Figure S7). As summarized in Fig. 1B, SI Appendix, and Table S1, CH_3_C(O)OO^•^ exhibited the highest reactivity toward naphthalene and NPX (*k* > 10^8^ M^−1^ s^−1^), followed by naphthol, 2,4,6-trimethyphenol (TMP), 2,4-HD, CINN, and tryptophan (*k* ∼ 10^7^ M^−1^ s^−1^). The reactivity with benzoic acid, bisphenol A, phenyl methyl sulfoxide (PMSO), methyl *p*-tolyl sulfide, ibuprofen (IBP), and diethyltoluamide (DEET) were too low to determine.

Overall, CH_3_C(O)OO^•^ preferentially reacts with naphthyl compounds (*k* ∼ 10^7^–10^8^ M^−1^ s^−1^), and, to a lesser extent, aromatic, and diene compounds (*k* ∼ 10^6^–10^7^ M^−1^ s^−1^) (Fig. 1B, SI Appendix, Table S1). Notably, N- and S-containing compounds, that are electron-rich and susceptible to oxidation by ^•^OH (46), ^•^Cl (46), chlorine (47), high-valent iron (48), or peroxyacids (49, 50), are not efficiently degraded by CH_3_C(O)OO^•^, distinguishing CH_3_C(O)OO^•^ from other reactive species in environmental processes. According to previous studies, CH_3_C(O)OO^•^ usually react with aromatic and diene compounds via radical addition to C=C double bonds, followed by CH_3_C(O)O^•^-leaving reaction, resulting in epoxidation reaction (Eq. 11) (3, 33, 45). On the other hand, CH_3_C(O)OO^•^ oxidize ABTS and TMPD via an electron transfer pathway, producing PAA and organic radical (ABTS^•+^ or TMPD^•+^) (8). The oxidation mechanism should be further elucidated by transient spectra or mass spectrometry techniques for oxidation products.


(11) $$X+CH_{3}C(O)OO^{∙}\to CH_{3}C(O)\mathrm{OO}-X^{∙}\to X-O+CH_{3}C(O)O^{∙}$$

The above reactivity information can shed light on the recent debates about reactive species in PAA-related AOPs. In delineating the reactive species in metal-PAA AOPs, the 100% conversion of sulfoxides (e.g. DMSO and PMSO) to sulfones has been frequently used as evidence for the absence of CH_3_C(O)OO^•^, and the oxidation of CBZ has been attributed to other species due to its “suspected low reactivity” with CH_3_C(O)OO^•^ (15, 23). Herein, we demonstrated that CH_3_C(O)OO^•^ reacts with CBZ at (3.0 ± 0.6) × 10^6^ M^−1^ s^−1^, hence holds the potential to degrade CBZ. On the contrary, given the negligible reactivity of CH_3_C(O)OO^•^ toward S-containing compounds, the transformation products of sulfoxides cannot be used as the evidence for the absence of CH_3_C(O)OO^•^.

### Reactive species in PR of acetaldehyde

**Fig. 4.**
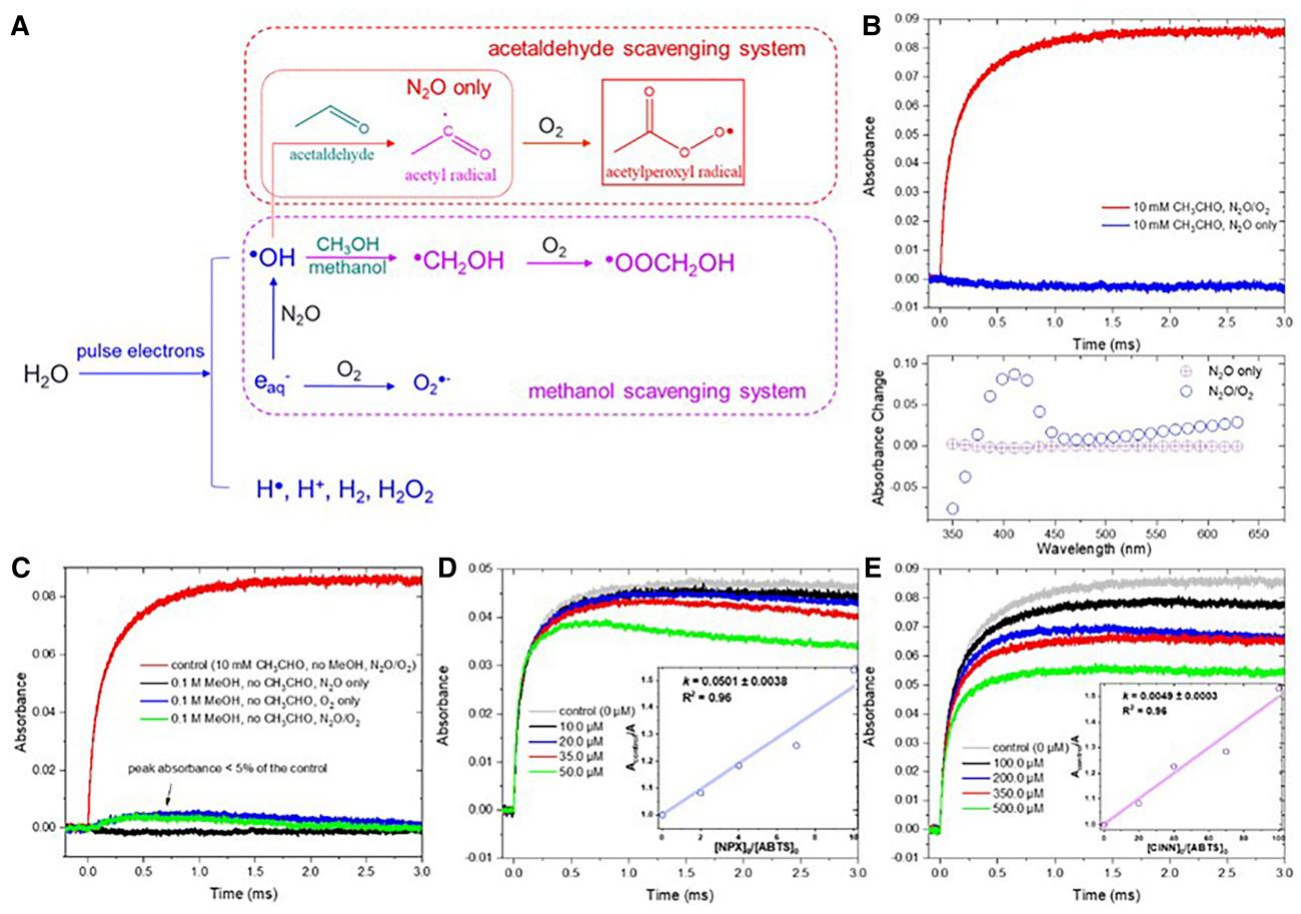
The mechanism for acetylperoxyl radical generation by PR of acetaldehyde (A), ABTS oxidation kinetics and transient absorption spectra changes (observed 1.0 ms after PR) in solutions saturated with N_2_O/O_2_ or N_2_O (B), effect of methanol (C), NPX (D), and *trans*-CINN (E) on ABTS oxidation during PR of acetaldehyde (represented by ABTS^•+^ formation recorded at 423 nm). Experimental conditions: [acetaldehyde] = 10 mM [for (B), (D), (E)], [ABTS] = 5 µM, pH_0_ = 5.7 (not buffered), radiation dose = 10.4 Gy [pulse = 4 ns, for (B), (C), (E)], or 16.5 Gy [pulse width = 5 ns, for (D)].

PR of acetaldehyde was applied as an alternative clean source of CH_3_C(O)OO^•^ to verify the rate constants (32). The absorbance spectrum of ABTS^•+^ obtained after PR was similar to that of LFP (Fig. 2B), indicating bleaching of the ground-state ABTS peak absorbance (∼340 nm) with the appearance of a transient absorbing species in the vicinity of 415 nm (Fig. 4B). As shown in Fig. 4B, the formation of ABTS^•+^ at 423 nm built up slowly within 1.0 ms and stayed stable in 3.0 ms.

In brief, the radiolysis of water leads to formation of hydrated electrons (e_aq_^−^), hydroxyl radicals (^•^OH), protons (H^+^), and a low yield of hydrogen atoms and molecular products (^•^H, H_2_O_2_, H_2_). In the presence of N_2_O (2.8 × 10^−2^ M), e_aq_^−^ and ^•^H will be converted to ^•^OH (32). Then, ^•^OH is rapidly scavenged by 10 mM of acetaldehyde through H-abstraction oxidation to generate CH_3_C(O)^•^, as the precursor for CH_3_C(O)OO^•^ in the presence of O_2_ (Fig. 4A). Notably, O_2_ could compete for e_aq_^−^ with N_2_O, generating HO_2_^•^/O_2_^•−^ radicals as another candidate reactive species (Fig. 4A). Overall, the reactive species that may contribute to ABTS oxidation during PR include ^•^OH, CH_3_C(O)^•^, CH_3_C(O)OO^•^, H_2_O_2_, and HO_2_^•^/O_2_^•−^.

We first tested PR of acetaldehyde with ABTS in an anoxic environment (N_2_O saturation only) and found that ABTS^•+^ transient generation was totally inhibited (Fig. 4B). The lack of O_2_ suppressed the formation of CH_3_C(O)OO^•^ and HO_2_^•^/O_2_^•−^, but could not reduce the yield of ^•^OH and CH_3_C(O)^•^. Therefore, the absence of ABTS^•+^ signal in the anoxic solution confirmed that (i) ^•^OH was completely scavenged by 10 mM of acetaldehyde hence could not lead to ABTS oxidation; and (ii) CH_3_C(O)^•^ was ineffective for ABTS oxidation, thus, its role in the LFP system and UV reactor should also be negligible, which was consistent with previous studies (32).

Furthermore, H_2_O_2_ itself is a relatively weak oxidant and we have confirmed that it could not react with ABTS (49). Finally, to evaluate the role of HO_2_^•^/O_2_^•−^, we used methanol, instead of acetaldehyde, as a scavenger of ^•^OH, while not affecting HO_2_^•^/O_2_^•−^ production from the reaction between O_2_ and e_aq_^−^ (Fig. 4A and C). As a result, we found that the ABTS^•+^ signal at 423 nm became minimal (<5% of the acetaldehyde experiments) regardless of the saturation gas, suggesting that the reaction between HO_2_^•^/O_2_^•−^ and ABTS, if any, should be negligible. Obviously, although ^•^OH-scavenging by methanol generates ^•^CH_2_OH and ^•^OOCH_2_OH radicals (Fig. 4A), they could not oxidize ABTS in the system (Fig. 4C). These results are consistent with the previous studies that reported the limited reactivity of carbon-centered radicals and alkyl peroxyl radicals, distinguishing CH_3_C(O)OO^•^ from other organic radicals in terms of reactivity (2, 4, 12).

### Competition kinetics study in PR of acetaldehyde

Then, PR of acetaldehyde was harnessed to verify the rate constants for two major probes in this study, i.e. NPX and CINN, using the competition kinetics approach (Eqs. 4-6). As shown in Fig. 4D and E, the rate constants for NPX and CINN were determined to be (1.0 ± 0.1) × 10^8^ and (9.8 ± 0.6) × 10^6^ M^−1^ s^−1^, respectively, by the PR approach (SI Appendix, Table S1). These data are in excellent agreement with those obtained from LFP, confirming the reliability of the LFP approach and the effectiveness of these probes for UV reactor experiments. The rate constants for TMP and cysteine were also studied by PR (Fig. 1B, SI Appendix, Table S1, Figure S6).

### Remodeling the reaction kinetics of UV/PAA

**Fig. 5.**
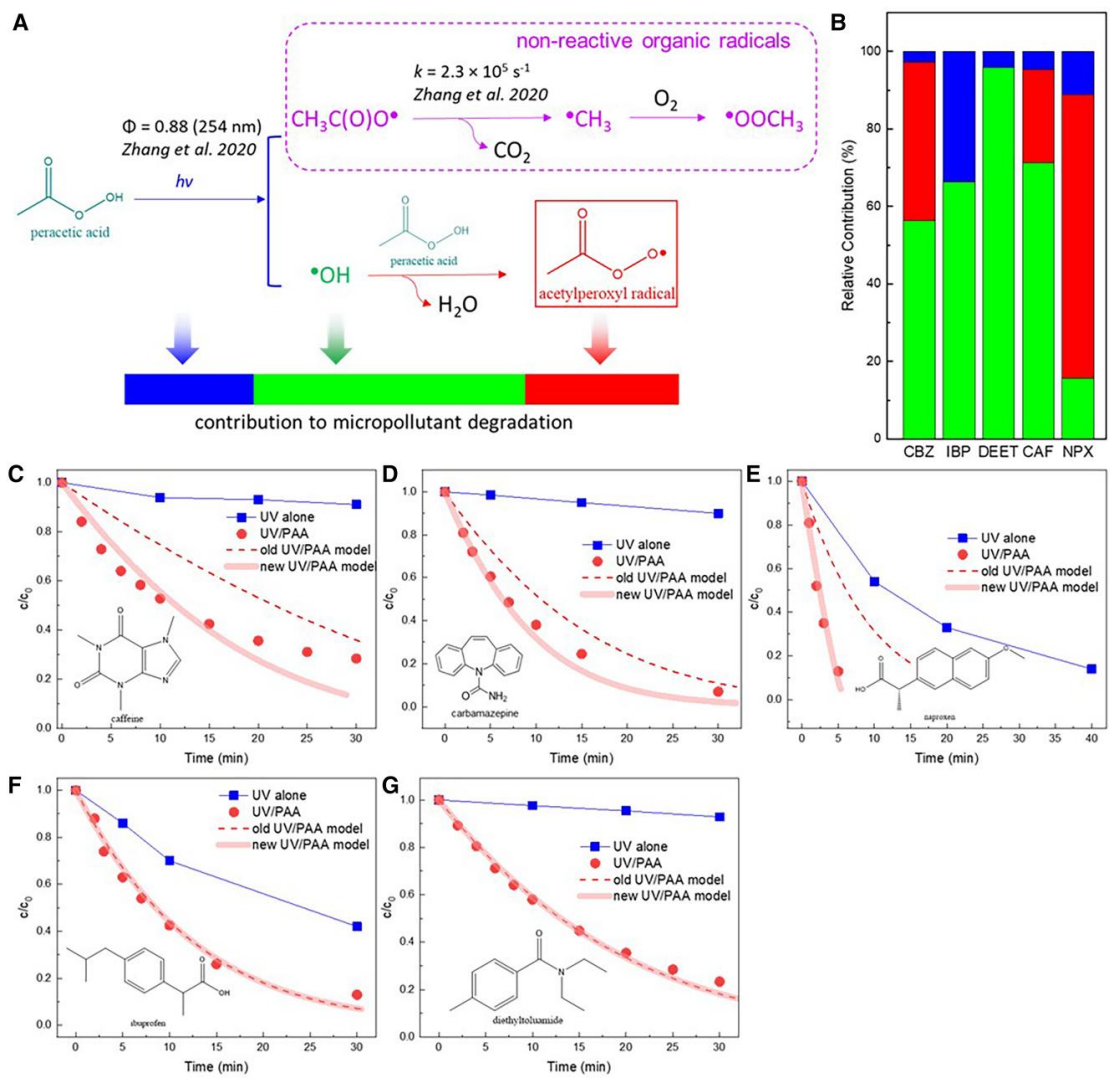
Mechanisms for acetylperoxyl radical generation by UV/PAA AOP (A), relative contribution of UV, ^•^OH, and CH_3_C(O)OO^•^ to organic compound degradation by UV/PAA (B), kinetic modeling of the degradation by UV/PAA (C-G). The experimental data are retrieved from our previous studies (Zhang and Huang; Cai *et al.*) with permission (12, 19). The new UV/PAA model included the reactions from the old model (12) and new reactions between CH_3_C(O)OO^•^ and organic compounds (SI Appendix, Tables S1 and S2).

This study is among the first to establish a large reactivity dataset for an organic radical (CH_3_C(O)OO^•^), which could be incorporated into the kinetic models for natural and engineered systems to enhance modeling accuracy. Herein, we take an established kinetic model on UV/PAA AOP as the example and demonstrate the importance of CH_3_C(O)OO^•^-related reactions. UV/PAA is an emerging (waste)water treatment technology that has been extensively applied for inactivation of pathogens (20, 51, 52) and degradation of micropollutants (12, 19) (Fig. 5A, SI Appendix, Table S2). In brief, photolysis of PAA by UV cleaves the O–O bond and generate ^•^OH and CH_3_C(O)O^•^, which in turn produce CH_3_C(O)OO^•^, ^•^OOCH_3_, and ^•^CH_3_ through radical chain-reactions. Unlike CH_3_C(O)O^•^ and ^•^CH_3_, whose reactivity and steady-state concentrations are too low to contribute to decontamination, the steady-state concentration of CH_3_C(O)OO^•^ is ∼3 orders of magnitude higher than ^•^OH, indicating its significant contribution in oxidation (12, 20). However, the contribution of CH_3_C(O)OO^•^ could not be included in the kinetic model previously due to lack of reliable reactivity information, leading to an underestimation of the overall oxidation efficiency. Herein, we integrated the reactions between CH_3_C(O)OO^•^ and organic contaminants into the UV/PAA kinetic model to simulate the removal of micropollutants and to quantify the relative contribution of direct UV photolysis, CH_3_C(O)OO^•^, and ^•^OH in the system (Fig. 5B). As shown in Fig. 5C-G, the incorporation of CH_3_C(O)OO^•^ oxidation reactions significantly improved the accuracy of modeling micropollutant degradation by UV/PAA. Although its contribution is negligible for IBP and DEET due to low reactivity, CH_3_C(O)OO^•^ accounted for 24.08, 40.78, and 73.04% removal of caffeine, CBZ, and NPX, respectively (Fig. 5B, SI Appendix, Table S3). These results further demonstrate the environmental significance of CH_3_C(O)OO^•^ and indirectly verify the rate constants generated in this study.

## Conclusions

Compared with the common inorganic radicals, the importance of ubiquitous organic radicals in the environment has been scarcely studied. In this study, we established systematic approaches for quantifying the reactivity of an organic peroxyl radical with high reactivity, i.e. CH_3_C(O)OO^•^. LFP of biacetyl, UV reactor with biacetyl, and PR of acetaldehyde under aerobic condition, were verified as clean sources of CH_3_C(O)OO^•^. The second-order rate constants between CH_3_C(O)OO^•^ and 34 different organic compounds demonstrated high reproducibility during cross-validation and literature comparison. CH_3_C(O)OO^•^ exhibited the highest reactivity toward naphthyl and diene compounds, while showed much less capacity in oxidizing N-, and S-containing functional groups. Even with such structural selectivity, the high reaction rates of CH_3_C(O)OO^•^ render its nonnegligible contribution in environmental processes. Indeed, incorporating the new kinetic information remarkably improved the modeling accuracy for UV/PAA AOP, where CH_3_C(O)OO^•^ contributes significantly to the oxidation of several common organic contaminants in water. The kinetic dataset and experimental approaches established by this study will be useful to facilitate future research on organic peroxyl radicals.

## Materials and methods

### Chemicals and reagents

The selected compounds for study are listed in SI Appendix, Table S1. The sources for these compounds and other chemicals are provided in SI Appendix.

### Laser flash photolysis

The setup of the LFP system has been illustrated in our previous studies and Fig. 2A (30). The reaction solution containing biacetyl (10 mM), ABTS, and a selected compound was pumped through the reaction cell that received pulse laser activation at λ = 351 nm. Produced ABTS^•+^ was measured, by a continuous wave laser at 635 nm as the light source combined with a fast photodiode as the detector (SI Appendix), over the reaction time after activation by the laser pulse.

### Spin-trapped EPR

EPR study was conducted with a Bruker EMX micro spectrometer and an ER 4103TM resonator to evaluate the radical species during biacetyl photolysis. DMPO was used as the trapping agent. The solution of biacetyl with DMPO (2 mM) was prepared in either water or TBA and irradiated by a 100 W mercury arc lamp that emitted light from 200 to 600 nm (connected by optical fiber, Fig. 3A), and the glass tube cut off any irradiation below 300 nm (SI Appendix).

### Degradation of selected compounds in the UV reactor

The setup of a collimated beam UV reactor was described in our previous studies (SI Appendix, Figure S7A) (20). A quartz reactor (20 mL), containing biacetyl (2 mM), probe (20 µM), one selected compound (20 µM), and phosphate buffer (pH 5.7, 5 mM) was irradiated under an low pressuer ultraviolet (LPUV) lamp at 254 nm. The reactor was open to the atmosphere with a surface to volume ratio at 0.83 cm^−1^ to ensure sufficient reaeration during the experiments. Periodically, 0.5-mL aliquots were collected to amber vials for concentration analysis using an Agilent 1100 high-performance liquid chromatography equipped with a diode-array detector. The experiments were conducted in duplicate.

### Pulse radiolysis

The PR experiments were performed using an 8 MeV Linear Accelerator at the Notre Dame Radiation Laboratory (SI Appendix) (53). The prepared solutions containing acetaldehyde, ABTS, and one organic compound, at pH 5.7. Saturation with N_2_O or N_2_O/O_2_ mixtures was achieved by bubbling for at least 25 min per 80 mL sample. Mixtures of N_2_O/O_2_ were calibrated using flow meters and maintained at the same level in all experiments. Solutions were prepared fresh and protected from adventitious exposure to room light. The radiation dose was 10.4 Gy per 4 ns pulse or 16 Gy per 5 ns pulse, as determined by the thiocyanate dosimeter. The competition kinetics experiments for each compound were conducted under the identical condition and provided consistent results. The transient kinetic analysis of the produced ABTS^•+^ was conducted at 423 nm.

### Kinetic modeling

The rate constants for reactions between CH_3_C(O)OO^•^ and organic contaminants are incorporated into the kinetic model for UV/PAA (SI Appendix, Table S2) (12), and the contaminant removal by UV/PAA was modeled using the Kintecus program 4.55.31.

## Supplementary Material

pgae330_Supplementary_Data

## Data Availability

All study data are included in the article and SI Appendix.

## Change History

October 3, 2024: The Supplementary Material has been updated to remove author responses to reviewer comments, which had been erroneously included due to a vendor error.
