# Supplementary material for: Unveiling the environmental significance of acetylperoxyl radical: Reactivity quantification and kinetic modeling
Source: PNAS Nexus. 2024 Aug 7;3(8):pgae330. doi: 10.1093/pnasnexus/pgae330 (PMC11346367; doi:10.1093/pnasnexus/pgae330)
Supplement: pgae330_Supplementary_Data [file pgae330_supplementary_data.docx]

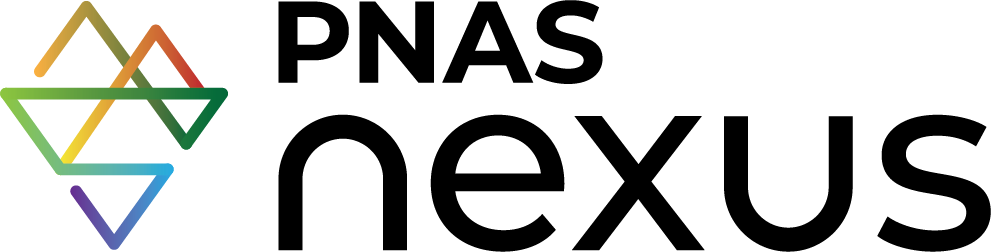


**Supporting Information for**

**Unveiling the environmental significance of acetylperoxyl radical: reactivity quantification and kinetic modeling**

Junyue Wang,^a^ Thomas Schaefer,^b^ Aliaksandra Lisouskaya,^c^ Daniele S. Firak,^b^

Xiaoyue Xin,^a^ Lingjun Meng,^a^ Hartmut Herrmann,^b^ Virender K. Sharma,^d^ Ching-Hua Huang*^,a^

^a^School of Civil and Environmental Engineering, Georgia Institute of Technology, 200 Bobby Dodd Way, Atlanta, Georgia 30332, USA

^b^Atmospheric Chemistry Department (ACD), Leibniz Institute for Tropospheric Research (TROPOS), Permoserstraße 15, 04318, Leipzig, Germany

^c^Radiation Laboratory, University of Notre Dame, 102 Radiation Research Building, Notre Dame, Indiana 46556, United States

^d^Department of Environment and Occupational Health, School of Public Health, Texas A&M University, 212 Adriance Lab Road, College Station, Texas 77843, United States

*Ching-Hua Huang, Ph.D. Professor

**Email:**  [ching-hua.huang@ce.gatech.edu](mailto:ching-hua.huang@ce.gatech.edu)

**This PDF file includes:**

Supporting text

Figures S1 to S7

Tables S1 to S3

SI References

Supporting Information Text

**Chemicals and Reagents.** Biacetyl, 2,2′-azino-bis(3-ethylbenzothiazoline-6-sulfonic acid) (ABTS), perchloric acid, sodium hydroxide, sodium phosphate, acetaldehyde, and the selected compounds listed in **Table S1** were obtained from Sigma-Aldrich or Thermo Fisher.

**Laser Flash Photolysis** **Experimental Procedures.** The sample flowed through the quartz glass reaction cell (V = 28 mL) with an optical path length of 3.5 cm at a flow rate of 8.2 ± 0.2 mL/s. Pulse laser (f = 1 Hz) at E = 63 ± 1 or 52 ± 1 mJ was used to activate the biacetyl to generate acetylperoxyl radical. Note that, each group of competition kinetics experiments was conducted on the same day with a stable energy output. Thus, the change in laser energy could not affect our conclusions. For each experiment, eight laser shots were taken and the kinetic data were averaged for analysis. The solution pH was adjusted and measured before LFP tests, but not buffered or monitored during the LFP experiments. Analysis afterwards found the solution pH change was < 0.5 unit after the LFP.

It should be noted that the ambient light could also activate biacetyl for ABTS oxidation. During the experiments, we turned off the lights in the lab and the solution was stable for at least 10 min. The ABTS^●+^ formation was confirmed by the absorption spectra change after laser excitation, and was quantified by the absorbance at 635 nm (**Fig. S2**).

**Electron Paramagnetic Resonance (EPR)** **Experimental Procedures.** To directly detect the acetylperoxyl radical with the condition simulating the laser flash photolysis, we conducted EPR analysis during photolysis of biacetyl under UVA irradiation. Spin-trapping experiments were performed in a Bruker EMX Plus spectrometer, using the resonator 4103TM, with the following settings: microwave frequency = 9.853 GHz, modulation amplitude = 1.00 G, magnetic field scan = 150 G, sweep time = 15 s, conversion time = 10 ms, time constant = 5.12 ms, 2 accumulations. Spectra were acquired in the field delay mode at 5 s scan delay. A 100 W mercury arc lamp (OSRAM) was applied as the *in-situ* light source, with a complete cut-off for wavelength below 280 nm by glass capillaries and an optic fiber accessory (Quantum Design) to deliver the light to the samples.

**UV Photoreactor** **Experimental Procedures.** The set-up of a collimated beam UV reactor was described in our previous studies.(1) A LPUV lamp emitting light at 254 nm was used as the light source. A quartz reactor (20 mL), containing biacetyl, the probe, and 20 µM of a selected compound, was irradiated with a path length of 1.2 cm. The reactor received UV light at (9.5 ± 0.5)×10^-7^ Einstein/(L∙s) (or 0.54 ± 0.03 mW/cm^2^, measured by KI/KIO_3_ actinometry).

Periodically, 0.5-mL aliquots were taken from the UV reactor into amber vials. The concentrations of the target compounds were analyzed using an Agilent 1100 high performance liquid chromatography equipped with an Agilent Zorbax SB–C18 column (2.1 × 150 mm, 5 μm) and a diode-array detector (HPLC-DAD) with flow rates at 0.2-0.6 mL/min. Acetonitrile and water, at the ratio of 40:50, 40:60 or 20: 80 (v/v), were used as the mobile phase. The compounds were analyzed by their absorbance at 210, 250 or 280 nm.

**Pulse Radiolysis** **Experimental Procedures.** The experiments were conducted at room temperature, where a consistent stream of sample solution passed through a quartz cell with an optical path length of 1 cm. The 1000 W xenon lamp employed to capture UV-visible transient absorption measurements within milliseconds time intervals. A multi-channel detection system recorded a series of 24 monochromatic kinetic signals across all input channels of six Tektronix oscilloscopes triggered synchronously, as extensively detailed in previous research.(2) For radiation dosimetry, thiocyanate dosimeter, 10 mM KSCN solutions saturated with N_2_O were employed; the value $G\varepsilon$ of (SCN)_2_^−●^ was taken as (5.2 ± 0.05) × 10^-4^ m^2^ J^-1^ with $\varepsilon_{{(SCN)}_{2}^{-\boldsymbol{\cdot}}}$= 7580 M^–1^ cm^–1^.

**Kinetic Modeling.** The contaminant degradation in UV/PAA was simulated on the Kintecus program 4.55.31, using the “-show -ig:mass -INT:5 ” command, with the reactions listed in **Table S2**. We first used the model to simulate the concentrations of hydroxyl and acetylperoxyl radicals, and obtained similar results as that in Zhang et al.’s MATLAB model.(3) The radical concentrations were then used in the calculation of contaminant degradation.

**Table S1**. Second-Order Rate Constants between Acetylperoxyl Radical and Selected Compounds

|  | Compound | *k* (M^-1^s^-1^) | Method or Ref |
| --- | --- | --- | --- |
| aromatic compounds | nitrobenzene (NB) | (1.8±0.4)×10^6^ | UV reactor |
|  | 2,4,6-trimethylphenol (TMP) | (1.7±0.4)×10^7^ | UV reactor |
|  |  | (4.8±0.0)×10^7^ | PR |
|  | aniline (ANI) | (3.0±0.6)×10^6^ | UV reactor |
|  | styrene | 3.0×10^6^ | (4) ^a^ |
|  | bisphenol A (BPA) | (2.8±0.5)×10^6^ | UV reactor |
|  | benzoic acid (BA) | < 2.0×10^5^ | UV reactor ^c^ |
| naphthyl  compounds | naproxen (NPX) | (1.2±0.6)×10^8^ | LFP |
|  |  | (1.0±0.1)×10^8^ | PR |
|  | naphthalene (NPL) | (3.6±1.2)×10^8^ | UV reactor |
|  | naphthol | (1.8±0.6)×10^7^ | UV reactor |
| alkenes | *cis*-2-octene | 2.1×10^6^ | (5) |
|  | cyclohexene | 2.1×10^6^ | (6) |
| dienes | β-carotene | (9.2±0.6)×10^8^ | (5) |
|  | norbornadiene | 1.8×10^7^ | (6) |
|  | *trans*-propenylbenzene | 4.2×10^7^ | (4) |
|  | *trans*-cinnamic acid (CINN) | (1.0±0.2)×10^7^ | LFP |
|  |  | (9.8±0.6)×10^6^ | PR |
|  | 2,4-hexadiene (2,4-HD) | (1.5±0.3)×10^7^ | LFP |
| alcohols | *tert*-butyl alcohol (TBA) | < 2.0×10^5^ | LFP ^b^ |
| amino acids | glycine | < 2.0×10^5^ | LFP |
|  | aspartic acid | < 2.0×10^5^ | LFP |
|  | leucine | < 2.0×10^5^ | LFP |
|  | cysteine (CYS) | (5.2±0.2)×10^6^ | PR |
|  | tryptophan (TRY) | (1.6±0.3)×10^7^ | UV reactor |
| sulfides | methyl *p*-tolyl sulfide (MTS) | < 1.0×10^5^ | UV reactor |
|  | diphenyl sulfide | < 1.0×10^5^ | (4) |
| sulfoxides | diphenyl sulfoxide | < 1.0×10^5^ | (4) |
|  | phenyl methyl sulfoxide (PMSO) | < 1.0×10^5^ | UV reactor |
|  | dimethyl sulfoxide (DMSO) | < 1.0×10^5^ | LFP |
| pharmaceuticals | caffeine (CAF) | (1.1±0.2)×10^6^ | UV reactor |
|  | diethyltoluamide (DEET) | < 3.0×10^5^ | UV reactor |
|  | carbamazepine (CBZ) | (3.0±0.6)×10^6^ | UV reactor |
|  | ibuprofen (IBP) | < 3.0×10^5^ | UV reactor |
| probes | 2,2′-azino-bis(3-ethylbenzothiazoline-6-sulfonic acid) (ABTS) | (2.0±0.1)×10^9^ | LFP |
|  | ascorbic acid (ASC) | (9.4±0.2)×10^8^ | LFP |

1. Since Sawaki et al. (4) only provided reaction rate relative to cyclohexene among various compounds, the quantitative rate constants here are calculated by the absolute rate constant of cyclohexene provided by Hoshino et al.(6)
2. We assumed that the rate constant detection limit is when the compound scavenges > 20% of CH_3_C(O)OO^●^ in the presence of ABTS (2.5 µM).
3. We assumed the detection limit of the pseudo-first order degradation in the UV reactor is 0.002 min^-1^.

**Table S2.** Reactions for Modeling the Kinetics of Micropollutant Degradation by UV/PAA

| No. Reaction | | Rate constant | Ref |
| --- | --- | --- | --- |
|  | **Photolysis^a^** |  |  |
| 1 | H_2_O_2_ → 2 HO^•^ | Φ = 0.50, ε = 18.7 M^-1^cm^-1^ | (3) |
| 2 | CH_3_C(O)OOH → CH_3_COO^•^ + HO^•^ | Φ = 0.88, ε = 10.0 M^-1^cm^-1^ | (3) |
|  | **Reactive Oxygen Species** |  |  |
| 3 | H^+^ + HO_2_^-^ → H_2_O_2_ | 5.00×10^10^ M^-1^ s^-1^ | (7) |
| 4 | H_2_O_2_ → H^+^ + HO_2_^-^ | 1.30×10^-1^ s^-1^ | (7) |
| 5 | H^+^ + OH^-^ → H_2_O | 1.00×10^11^ M^-1^ s^-1^ | (7) |
| 6 | H_2_O → H^+^ + OH^-^ | 1.00×10^-3^ s^-1^ | (7) |
| 7 | H^+^ + O_2_^•-^ → HO_2_^•^ | 5.00×10^10^ M^-1^ s^-1^ | (7) |
| 8 | HO_2_^•^ → H^+^ + O_2_^•-^ | 7.00×10^5^ s^-1^ | (7) |
| 9 | HO^•^ + HO^•^ → H_2_O_2_ | 5.50×10^9^ M^-1^ s^-1^ | (7) |
| 10 | HO^•^ + OH- →O^•-^ + H_2_O | 1.30×10^10^ M^-1^ s^-1^ | (7) |
| 11 | HO^•^ + H_2_O_2_ → HO_2_^•^ + H_2_O | 2.70×10^7^ M^-1^ s^-1^ | (7) |
| 12 | HO^•^ + HO_2_^-^ → HO_2_^•^ + OH^-^ | 7.50×10^9^ M^-1^ s^-1^ | (7) |
| 13 | HO^•^ + HO_2_^•^ → O_2_ + H_2_O | 7.10×10^9^ M^-1^ s^-1^ | (7) |
| 14 | HO^•^ + O_2_^•-^ → O_2_ + OH^-^ | 1.00×10^10^ M^-1^ s^-1^ | (7) |
| 15 | HO_2_^•^ + HO_2_^•^ → H_2_O_2_ + O_2_ | 8.30×10^9^ M^-1^ s^-1^ | (7) |
| 16 | HO_2_^•^ + O_2_^•-^ → HO_2_^-^ + O_2_ | 9.70×10^7^ M^-1^ s^-1^ | (7) |
| 17 | HO_2_^•^ + H_2_O_2_ → O_2_ + HO^•^ +H_2_O | 3.00 M^-1^ s^-1^ | (7) |
| 18 | O_2_^•-^ + H_2_O_2_ → O_2_ + HO^•^ + OH^-^ | 1.30×10^-1^ M^-1^ s^-1^ | (7) |
| 19 | O^•-^ + H_2_O → HO^•^ + OH^-^ | 1.80×10^6^ M^-1^ s^-1^ | (7) |
| 20 | O^•-^ + O^•-^ → O_2_^2-^ | 4.65×10^9^ M^-1^ s^-1^ | (8) |
|  | **Organic Radicals** |  |  |
| 21 | CH_3_C(O)OH + HO^•^ → ^•^CH_2_C(O)OH + H_2_O | 1.60×10^7^ M^-1^ s^-1^ | (3) |
| 22 | CH_3_C(O)O⁻ + HO^•^ → ^•^CH_2_C(O)O⁻ + H_2_O | 8.50×10^7^ M^-1^ s^-1^ | (3) |
| 23 | CH_2_C(O)O⁻ + O_2_ → ^•^OOCH_2_C(O)O⁻ | 1.70×10^9^ M^-1^ s^-1^ | (3) |
| 24 | 2^•^OOCH_2_C(O)O⁻ →HOCC(O)O^-^+ HOCH_2_C(O)O^-^ + O_2_ | 2.20×10^7^ M^-1^ s^-1^ | (3) |
| 25 | 2 ^•^OOCH_2_C(O)O⁻ → 2 HOCC(O)O^-^ + H_2_O_2_ | 2.30×10^7^ M^-1^ s^-1^ | (3) |
| 26 | 2 ^•^OOCH_2_C(O)O⁻ → 2 ^•^OCH_2_C(O)O⁻ + O_2_ | 3.00×10^7^ M^-1^ s^-1^ | (3) |
| 27 | ^•^OOCH_2_C(O)O⁻ + HO_2_^•^→ HOCH_2_C(O)O⁻ + ^3^O + ^3^O_2_ | 9.80×10^6^ M^-1^ s^-1^ | (3) |
| 28 | 2^•^OCH_2_C(O)O⁻ → HOCH_2_C(O)O⁻ + HOCC(O)O⁻ | 7.30×10^9^ M^-1^ s^-1^ | (3) |
| 29 | ^•^OCH_2_C(O)O⁻ → HCHO + ^•^C(O)O⁻ | 1.00×10^6^ s^-1^ | (3) |
| 30 | ^•^C(O)O⁻ + O_2_ → CO_2_ + ^•^O_2_⁻ | 2.40×10^9^ M^-1^ s^-1^ | (3) |
| 31 | HOCC(O)O⁻ + HO^•^ → ^•^OCC(O)O⁻ + H_2_O | 2.60×10^9^ M^-1^ s^-1^ | (3) |
| 32 | HOCH_2_C(O)O⁻ + HO^•^ → HO^•^CHC(O)O⁻ + H_2_O | 8.60×10^8^ M^-1^ s^-1^ | (3) |
| 33 | HCHO + HO^•^ → products | 1.00×10^9^ M^-1^ s^-1^ | (3) |
| 34 | CH_3_C(O)OOH + HO^•^ → CH_3_C(O)OO^•^ + H_2_O | 1.30×10^9^ M^-1^ s^-1^ | (3) |
| 35 | CH_3_C(O)OOH+CH_3_C(O)O^•^→CH_3_C(O)OO^•^+ CH_3_C(O)O^-^ | 1.00×10^7^ M^-1^ s^-1^ | (3) |
| 36 | CH_3_C(O)OOH + HO_2_^•^ → CH_3_C(O)OO^•^ + H_2_O_2_ | 2.00×10^2^ M^-1^ s^-1^ | (3) |
| 37 | CH_3_C(O)OO^•^ + CH_3_C(O)OO^•^ → 2 CH_3_C(O)O^•^ + O_2_ | 8.30×10^9^ M^-1^ s^-1^ | (3) |
| 38 | CH_3_C(O)OO^•^ + HO_2_^•^ → CH_3_C(O)OH + ^3^O + ^3^O_2_ | 2.00×10^6^ M^-1^ s^-1^ | (3) |
| 39 | CH_3_C(O)OO^•^ → HO_2_^•^ + CH_2_CO | 1.82 s^-1^ | (3) |
| 40 | CH_2_CO + H_2_O → CH_3_C(O)OH | 44.00 s^-1^ | (3) |
| 41 | CH_3_C(O)O^•^ + CH_3_C(O)O^•^ → (CH_3_C(O)O)_2_ | 1.00×10^9^ M^-1^ s^-1^ | (3) |
| 42 | CH_3_C(O)O^•^ → ^•^CH_3_ + CO_2_ | 2.30×10^5^ s^-1^ | (3) |
| 43 | ^•^CH_3_ + O_2_ → ^•^OOCH_3_ | 4.70×10^9^ M^-1^ s^-1^ | (3) |
| 44 | ^•^OOCH_3_ + ^•^OOCH_3_ → HCHO + CH_3_OH + O_2_ | 2.80×10^8^ M^-1^ s^-1^ | (3) |
| 45 | ^•^OOCH_3_ + ^•^OOCH_3_ → 2 HCHO + H_2_O_2_ | 2.50×10^7^ M^-1^ s^-1^ | (3) |
| 46 | ^•^OOCH_3_ + ^•^OOCH_3_ → 2 ^•^OCH_3_ + O_2_ | 2.20×10^8^ M^-1^ s^-1^ | (3) |
| 47 | ^•^OOCH_3_ + HO_2_^•^ → CH_3_OH + ^3^O + ^3^O_2_ | 5.00×10^8^ M^-1^ s^-1^ | (3) |
| 48 | ^•^OCH_3_ →^•^CH_2_OH | 5.00×10^5^ s^-1^ | (3) |
| 49 | ^•^CH_2_OH + O_2_ → OHCH_2_OO^•^ | 4.90×10^9^ M^-1^ s^-1^ | (3) |
| 50 | OHCH_2_OO^•^ → HCHO + HO_2_^•^ | 10.00 s^-1^ | (3) |
| 51 | OHCH_2_OO^•^ + OH⁻ → HCHO + O_2_^•^⁻ + H_2_O | 1.50×10^10^ M^-1^ s^-1^ | (3) |
| 52 | OHCH_2_OO^•^ + OHCH_2_OO^•^ → 2 OHCH_2_O^•^ + O_2_ | 8.90×10^8^ M^-1^ s^-1^ | (3) |
| 53 | OHCH_2_OO^•^ + OHCH_2_OO^•^ → 2 HCOOH + H_2_O_2_ | 1.60×10^9^ M^-1^ s^-1^ | (3) |
| 54 | OHCH_2_OO^•^ + HO_2_^•^ → CH_2_(OH)_2_ + ^3^O + O_2_ | 2.00×10^6^ M^-1^ s^-1^ | (3) |
| 55b | CH_3_OH + HO^•^→^•^CH_2_OH (93%) + ^•^OCH_3_ (7%) + H_2_O | 9.70×10^8^ M^-1^ s^-1^ | (3) |
|  | **Phosphate Buffer** |  |  |
| 56 | H_2_PO_4_^-^ + H^+^ → H_3_PO_4_ | 5.00×10^10^ M^-1^ s^-1^ | (7) |
| 57 | H_3_PO_4_ → H_2_PO_4_^-^ + H^+^ | 3.87×10^8^ s^-1^ | (7) |
| 58 | HPO_4_^2-^ + H^+^ → H_2_PO_4_^-^ | 5.00×10^10^ M^-1^ s^-1^ | (7) |
| 59 | H_2_PO_4_^-^ → HPO_4_^2-^ + H^+^ | 3.15×10^3^ s^-1^ | (7) |
| 60 | PO4^3-^ + H^+^ → HPO_4_^2-^ | 5.00×10^10^ M^-1^ s^-1^ | (7) |
| 61 | HPO_4_^2-^ → PO_4_^3-^ + H^+^ | 2.50×10^-2^ s^-1^ | (7) |
| 62 | HO^•^ + HPO_4_^2-^ → HPO_4_^•-^ + OH^-^ | 1.50×10^5^ M^-1^ s^-1^ | (7) |
| 63 | HO^•^ + H_2_PO_4_^-^ → HPO_4_^•-^ + H_2_O | 2.00×10^4^ M^-1^ s^-1^ | (7) |
| 64 | H_2_O_2_ + HPO_4_^•-^ → H_2_PO_4_^-^ + HO_2_^•^ | 2.70×10^7^ M^-1^ s^-1^ | (7) |
| 65 | HO^•^ + H_3_PO_4_ → H_2_PO_4_^•^ + H_2_O | 1.37×10^6^ M^-1^s^-1^ | (8) |
| 66 | H_2_O_2_ + H_2_PO_4_^•+^ → H_2_PO_4_^-^ + 2 H^+^ + O_2_^•-^ | 5.50×10^7^ M^-1^s^-1^ | (8) |
| 67 | O^•-^ + HPO_4_^2-^ → products | 3.50×10^6^ M^-1^s^-1^ | (7) |
| 68 | H_2_O + H_2_PO_4_^•^ → H_3_PO_4_ + HO^•^ | 1.30×10^5^ M^-1^s^-1^ | (7) |
|  | **Organic Contaminants ^b^** |  |  |
| 69 | CAF + HO^•^ → products | 6.40×10^9^ M^-1^ s^-1^ | (9) |
| 70 | IBP + HO^•^ → products | 7.07×10^9^ M^-1^ s^-1^ | (10) |
| 71 | NPX + HO^•^ → products | 8.61×10^9^ M^-1^ s^-1^ | (10) |
| 72 | CBZ + HO^•^ → products | 8.80×10^9^ M^-1^ s^-1^ | (11) |
| 73 | DEET + HO^•^ → products | 6.70×10^9^ M^-1^ s^-1^ | (9) |
| 74 | CAF + CH_3_C(O)OO^•^ → products | See **Table S1** | this  study |
| 75 | IBP + CH_3_C(O)OO^•^ → products |  |  |
| 76 | NPX + CH_3_C(O)OO^•^ → products |  |  |
| 77 | CBZ + CH_3_C(O)OO^•^ → products |  |  |
| 78 | DEET + CH_3_C(O)OO^•^ → products |  |  |

1. The values at 254 nm was provided.
2. The direct photolysis was included in our modeling but the rate constants are dependent on the light source and reactor set-up hence not included in this table.

**Table S3. Calculation of Relative Contribution of Direct Photolysis and Radicals**

|  | steady-state concentration | | pseudo-first-order degradation | | | relative contribution | | |
| --- | --- | --- | --- | --- | --- | --- | --- | --- |
|  | [^●^OH]_ss_  (10^-13^ M) | [CH_3_C(O)OO^●^]_ss_  (10^-10^ M) | *k*_OH_  (s^-1^) | *k*_CH3C(O)OO●_  (s^-1^) | *k*_UV_ ^a^  (10^-5^ s^-1^) | ^●^OH  (%) | CH_3_C(O)OO^●^ (%) | UV  (%) |
| CBZ | 1.32 | 2.80 | 0.001162 | 0.00084 | 5.83 | 56.39 | 40.78 | 2.83 |
| IBP | 1.41 | 2.90 | 0.000947 | 0 | 48.02 | 66.37 | 0 | 33.61 |
| DEET | 1.34 | 2.82 | 0.000938 | 0 | 4.00 | 95.91 | 0 | 4.09 |
| CAF | 1.41 | 2.78 | 0.000905 | 0.00031 | 5.83 | 71.32 | 24.08 | 4.59 |
| NPX | 1.38 | 0.39 | 0.001186 | 0.005518 | 85.03 | 15.70 | 73.04 | 11.31 |

1. The photolysis rates represent the conditions in Cai et al. (11) and Zhang et al. (3) studies, which are different from this study (**Fig. S7**).

**Fig. S1**. The change of absorption spectra after laser flash photolysis of biacetyl (10 mM) and ABTS (2.5 µM) solution. Control data was taken by flowing the solution through the reaction cell without laser activation, and overlapped with the original spectra (black line).

**Fig. S2**. The extinction coefficient for ABTS^●+^. As ABTS’s absorption at > 400 nm is negligible, the absorption spectra from 400-800 nm after ABTS oxidation was all attributed to ABTS^●+^, and was normalized based on its reported absorption at 415 nm (i.e., ε_415_ = 3.4×10^4^ M^-1^cm^-1^)(12). Therefore, the absorbance at 635 nm could be utilize to indicate ABTS^●+^ formation during LFP experiments.

**Fig. S3**. Effects of pH and laser energy on ABTS oxidation by LFP of biacetyl (represented by ABTS^●+^ formation recorded at 635 nm). Experimental conditions: [biacetyl]_0_ = 10 mM, [ABTS]_0_ = 2.5 µM. Note that the same blank data was used in **Figs**. **2, S3, S4, S5** where the same conditions were applied.


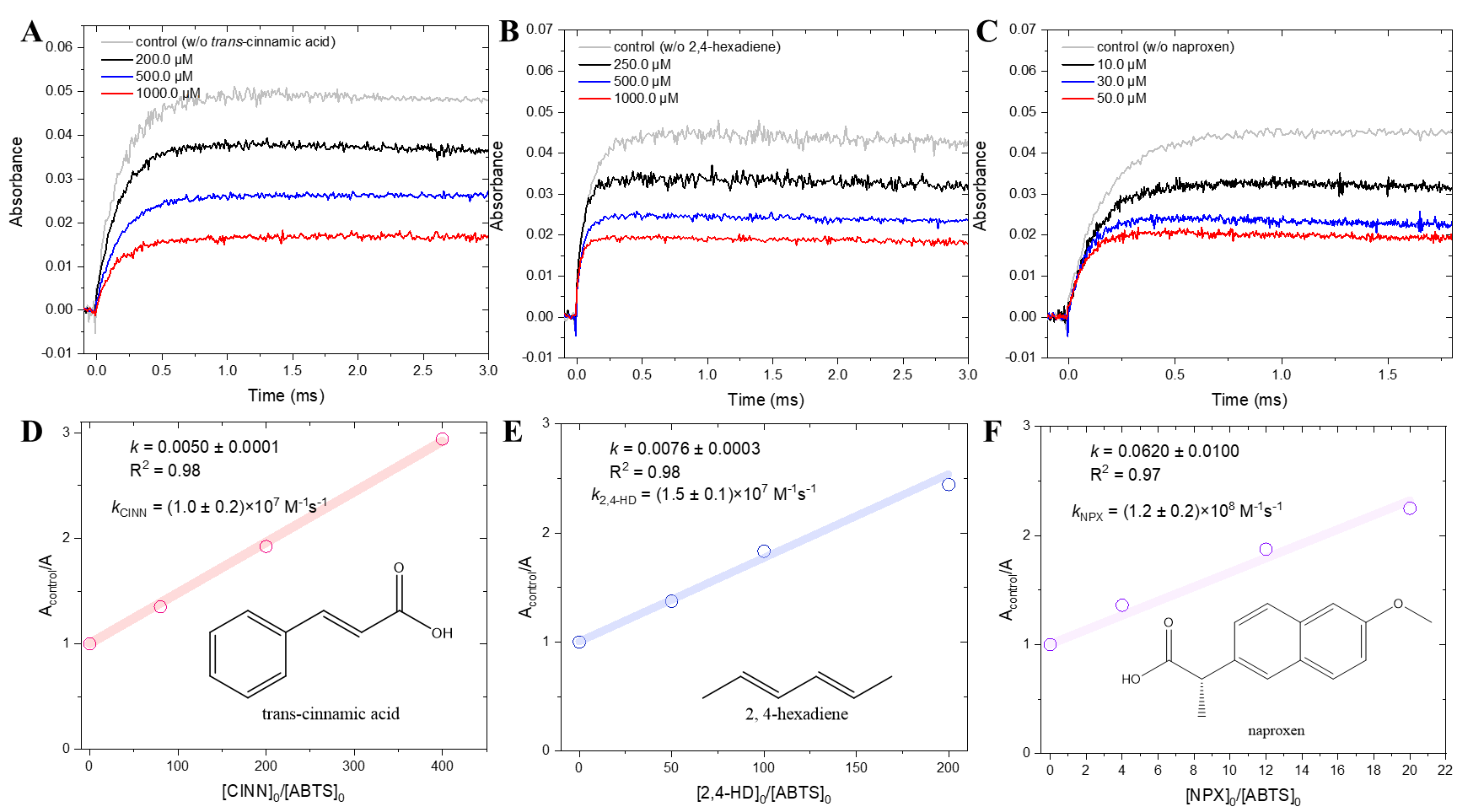


**Fig. S4**. Effects of the concentration of *trans*-cinnamic acid (A), 2,4-hexadiene (B), and naproxen (C) on ABTS oxidation by LFP of biacetyl (represented by ABTS^●+^ formation recorded at 635 nm); linear relationship between competitor/ABTS molar ratio and relative ABTS^●+^ formation (D-F). Experimental conditions: [biacetyl]_0_ = 10 mM, pH = 5.7, no buffer; for *trans*-cinnamic acid: [ABTS]_0_ = 2.5 µM, laser energy = 63 ± 1 mJ; for 2,4-hexadiene: [ABTS]_0_ = 5.0 µM, laser energy = 63 ± 1 mJ; for naproxen: [ABTS]_0_ = 2.5 µM, laser energy = 52 ± 1 mJ. Note that the same blank data was used in **Figs**. **2, S3, S4, S5** where the same conditions were applied.

**Fig. S5**. Effects of competitive compounds on ABTS oxidation by LFP of biacetyl (represented by ABTS^●+^ formation recorded at 635 nm). Experimental conditions: [biacetyl]_0_ = 10 mM, [ABTS]_0_ = 2.5 µM, laser energy = 63 ± 1 mJ. Note that the same blank data was used in **Figs**. **2, S3, S4, S5** where the same conditions were applied.


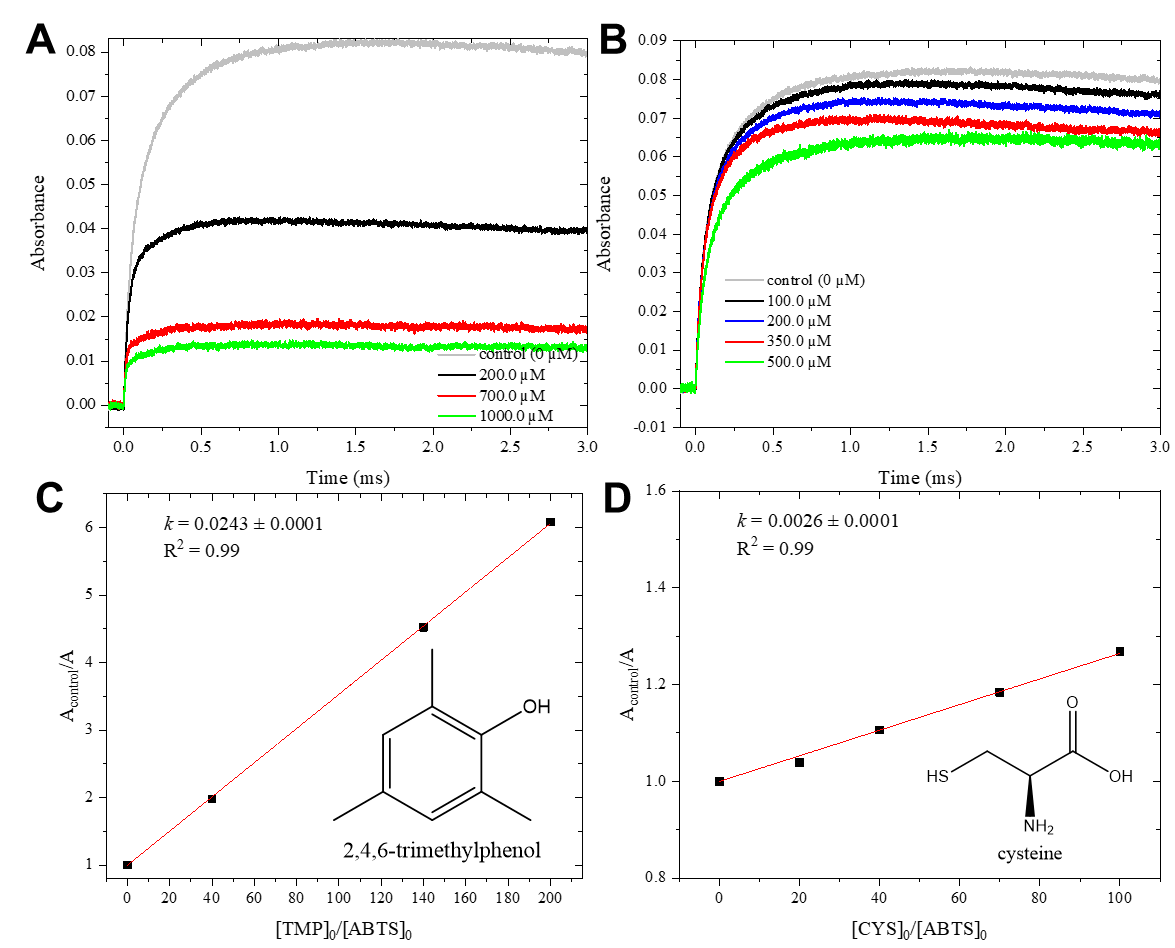


**Fig. S6**. Effect of 2,4,6-trimethylphenol (A, C) and cysteine (B, D) on ABTS oxidation by PR of acetaldehyde (represented by ABTS^●+^ formation recorded at 423 nm). Experimental conditions: [acetaldehyde]_0_ = 10 mM, [ABTS]_0_ = 5 µM, pH_0_ = 5.7 (not buffered), radiation dose = 10.6 Gy (pulse width = 4 ns). Note that the same blank data was used in **Figs**. **4, S6** where the same conditions were applied.


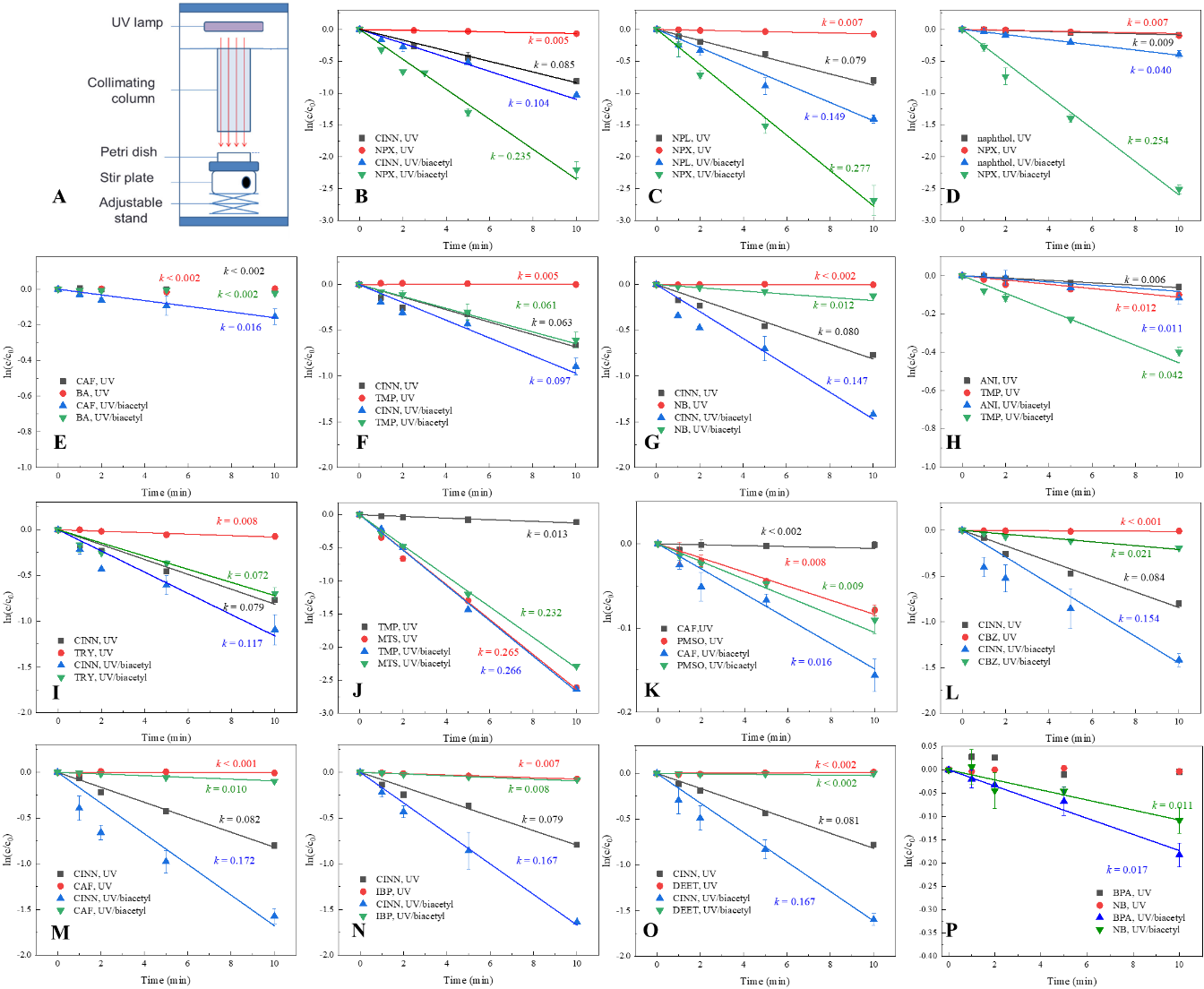


**Fig. S7**. The set-up of UV reactor (A), and the degradation of organic contaminants by UV and UV/biacetyl processes (B-P). Experimental conditions: [biacetyl]_0_ = 2 mM, pH_0_ = 5.7, [phosphate buffer] = 5 mM, [organic compounds]_0_ = 20 µM. For each experiment, one probe compound with known rate constant with CH_3_C(O)OO^●^ and one select compounds (rate constant unknown) were added together. The abbreviations of compounds are provided in **Table S1**. Error bars represent standard deviation between duplicate experiments.

**SI References:**

1. T. Zhang *et al.*, Inactivation of Bacteria by Peracetic Acid Combined with Ultraviolet Irradiation: Mechanism and Optimization. *Environ Sci Technol* **54**, 9652-9661 (2020).

2. A. Lisovskaya, K. Kanjana, D. M. Bartels, One-electron redox kinetics of aqueous transition metal couples Zn(2+/+), Co(2+/+), and Ni(2+/+) using pulse radiolysis. *Phys Chem Chem Phys* **22**, 19046-19058 (2020).

3. T. Zhang, C.-H. Huang, Modeling the Kinetics of UV/Peracetic Acid Advanced Oxidation Process. *Environ Sci Technol* **54**, 7579-7590 (2020).

4. Y. Sawaki, Y. Ogata, Reactivities of acylperoxy radicals in the photoreaction of .alpha.-diketones and oxygen. *J Org Chem* **49**, 3344-3349 (1984).

5. A. Mortensen, Scavenging of acetylperoxyl radicals and quenching of triplet diacetyl by β-carotene: mechanisms and kinetics. *J Photochem and Photobiol B* **61**, 62-67 (2001).

6. M. Hoshino, M. Kagata, H. Seki, H. Seto, Studies of Acetylperoxy Radicals Produced by Photolysis of Biacetyl in Aerated Benzene Solutions. Ion-Pair Formation with Tetramethyl-p-phenylenediamine. *J Am Chem Soc* **118**, 2160-2165 (1996).

7. K. Guo *et al.*, Comparison of the UV/chlorine and UV/H_2_O_2_ processes in the degradation of PPCPs in simulated drinking water and wastewater: Kinetics, radical mechanism and energy requirements. *Water Res* **147**, 184-194 (2018).

8. D. M. Bulman, S. P. Mezyk, C. K. Remucal, The Impact of pH and Irradiation Wavelength on the Production of Reactive Oxidants during Chlorine Photolysis. *Environ Sci Technol* **53**, 4450-4459 (2019).

9. P. Sun, W. N. Lee, R. Zhang, C.-H. Huang, Degradation of DEET and Caffeine under UV/Chlorine and Simulated Sunlight/Chlorine Conditions. *Environ Sci Technol* **50**, 13265-13273 (2016).

10. Y. Lei, S. Cheng, N. Luo, X. Yang, T. An, Rate Constants and Mechanisms of the Reactions of Cl(*) and Cl_2_(*-) with Trace Organic Contaminants. *Environ Sci Technol* **53**, 11170-11182 (2019).

11. M. Cai, P. Sun, L. Zhang, C.-H. Huang, UV/Peracetic Acid for Degradation of Pharmaceuticals and Reactive Species Evaluation. *Environ Sci Technol* **51**, 14217-14224 (2017).

12. Y. Lee, R. Kissner, U. von Gunten, Reaction of ferrate(VI) with ABTS and self-decay of ferrate(VI): kinetics and mechanisms. *Environ Sci Technol* **48**, 5154-5162 (2014).
